# Novel pathogenic variants of *DNAH5* associated with clinical and genetic spectra of primary ciliary dyskinesia in an Arab population

**DOI:** 10.3389/fgene.2024.1396797

**Published:** 2024-07-10

**Authors:** Dalal A. Al-Mutairi, Basel H. Alsabah, Petra Pennekamp, Heymut Omran

**Affiliations:** ^1^ Department of Pathology, Faculty of Medicine, Kuwait University, Kuwait City, Kuwait; ^2^ Zain Hospital for Ear, Nose and Throat, Kuwait City, Kuwait; ^3^ Department of Pediatrics, University Hospital Muenster, Muenster, Germany

**Keywords:** primary ciliary dyskinesia, pulmonary diseases, genetics of ciliopathy, *DNAH5* gene, laterality defects

## Abstract

**Introduction:** Primary ciliary dyskinesia (PCD) is caused by the dysfunction of motile cilia resulting in insufficient mucociliary clearance of the lungs. This study aimed to map novel PCD variants and determine their pathogenicity in PCD patients in Kuwait.

**Methods:** Herein, we present five PCD individuals belonging to a cohort of 105 PCD individuals recruited from different hospitals in Kuwait. Genomic DNAs from the family members were analysed to screen for pathogenic PCD variants. Transmission electron microscopy (TEM) and immunofluorescence (IF) analyses were performed on the nasal biopsies to detect specific structural abnormalities within the ciliated cells.

**Results:** Genetic screening and functional analyses confirmed that the five PCD individuals carried novel pathogenic variants of *DNAH5* causing PCD in three Arabic families. Of these, one multiplex family with two affected individuals showed two novel homozygous missense variants in *DNAH5* causing PCD with situs inversus; another multiplex family with two affected individuals showed two newly identified compound heterozygous variants in *DNAH5* causing PCD with situs solitus. In addition, novel heterozygous variants were identified in a child with PCD and situs solitus from a singleton family with unrelated parents. TEM analysis demonstrated the lack of outer dynein arms (ODAs) in all analysed samples, and IF analysis confirmed the absence of the dynein arm component of DNAH5 from the ciliary axoneme.

**Conclusion:** The newly identified pathogenic variants of *DNAH5* are associated with PCD as well as variable pulmonary clinical manifestations in Arabic families.

## Graphical Abstract

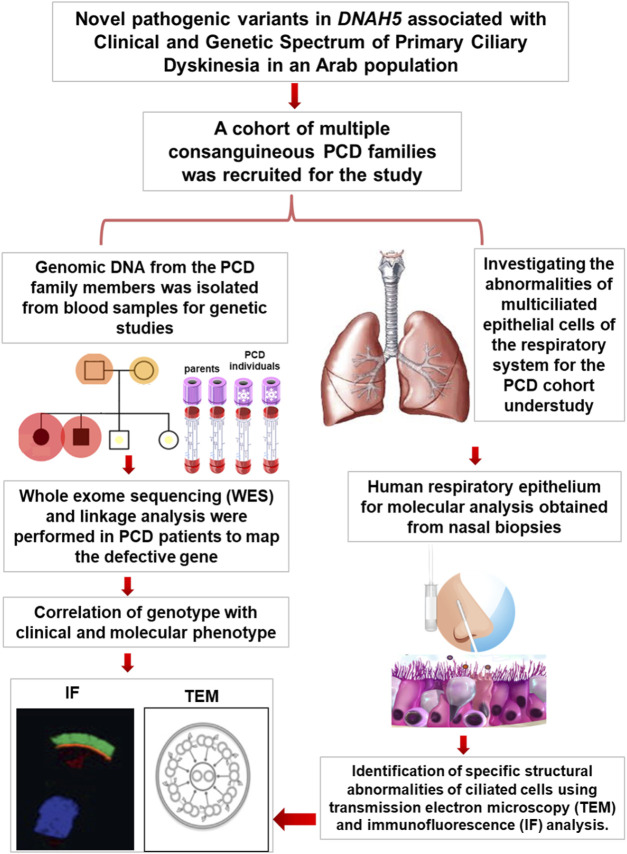

## Introduction

Primary ciliary dyskinesia (PCD; OMIM: 244400) is a clinically and genetically heterogeneous group of disorders of the motile cilia that results in abnormal mucociliary clearance and pulmonary dysfunction. PCD-affected individuals have a history of neonatal respiratory distress and suffer from lifelong symptoms of wet cough, rhinosinusitis, and otitis media. Recurrent chest infections are also common, which eventually lead to bronchiectasis and progressive decline in pulmonary functions (Ratjen et al., 2016; Davis et al., 2019; Al-Mutairi et al., 2022). Almost 50% of PCD affected individuals also have laterality defects, mainly situs inversus totalis and other situs abnormalities, owing to dysmotility of the monocilia of the left-right organiser (LRO). Male infertility and female subfertility are also associated with PCD (Zariwala et al., 2007; Leigh et al., 2009; Zariwala et al., 2011; Al-Mutairi et al., 2022). A recent study showed that the global incidence of PCD is 1 in 7,550 and that the frequency of PCD is higher in isolated populations, such as individuals of African descent, than in most other populations (Hannah et al., 2022; Al-Mutairi et al., 2023). Geographically isolated populations may also exhibit increased incidence of PCD owing to the practise of consanguineous marriages over multiple generations (Zariwala et al., 2007; Al-Mutairi et al., 2022; Hannah et al., 2022; Al-Mutairi et al., 2023), which is typical in the Kuwaiti population. Despite recent improvements in PCD diagnosis since the first reported case at the beginning of the 20th century, only a limited number of individuals have been accurately diagnosed with PCD, reflecting the limited capability of PCD diagnosis. Almost one-third of such individuals with well-characterised PCD phenotypes have no detectable genetic variants corresponding to known, published PCD-causing genetic variants (Knowles et al., 2013; Horani et al., 2016; Leigh et al., 2019; Wheway et al., 2021; Al-Mutairi et al., 2023). The diagnosis of PCD is therefore quite challenging, and a large proportion of PCD individuals are either misdiagnosed or receive delayed diagnoses since there is no ‘optimum PCD diagnostic approach’. In addition, some PCD-causing variants, especially compound heterozygous variants in PCD individuals with situs solitus, escape inclusion in the current diagnostic criteria for confirmed PCD diagnosis (Dalrymple and Kenia, 2019; Muller et al., 2021; Al-Mutairi et al., 2022; Goutaki and Shoemark, 2022; Al-Mutairi et al., 2023).

Currently, more than 53 PCD genes (OMIM: PS244400) have been identified that mostly encode structural components of the axonemes of motile cilia and sperm flagella (Al-Mutairi et al., 2022). In general, there are six structural genes that encode the dynein components reported to cause PCD and are associated with outer dynein arm (ODA) defects. Three ciliary structural genes, namely, *DNAH5* (Olbrich et al., 2002; Hornef et al., 2006; Failly et al., 2009), *DNAH9* (Loges et al., 2018), and *DNAH11* (Schwabe et al., 2008; Pifferi et al., 2010; Knowles et al., 2012), encode the axonemal ODA heavy chains. In addition, there are two ciliary structural genes *DNAI1* (Zariwala et al., 2006) and *DNAI2* (Loges et al., 2008; Al-Mutairi et al., 2022) that encode the axonemal ODA intermediate chains. Only one ciliary structural gene is known to encode the axonemal ODA light chains: *DNAL1* (Mazor et al., 2011). Dynein axonemal heavy chain 5 (DNAH5) (MIM: 603335) was the first reported gene to cause PCD associated with the absence of the ODAs; it is also the most common defective gene in PCD-affected Caucasians (Failly et al., 2009).

The purpose of this study was to determine the genetic causes and ciliary abnormalities of the respiratory system in Arabic PCD patients in Kuwait. This report summarises the genetic and functional analyses of ciliated respiratory epithelial cells from five PCD individuals with variable laterality defects who inherited novel pathogenic variants of *DNAH5*. Immunofluorescence (IF) and transmission electron microscopy (TEM) analyses show the complete absence of the dynein arm protein component DNAH5 as well as the absence of the axonemal ODAs in all five PCD individuals. This confirms that these newly reported variants of *DNAH5* are pathogenic and associated with PCD.

## Methods

### Inclusion criteria for the selected human subjects

Ethical approval for this project was obtained from the Ministry of Health Research Ethics Committee (Ethics ID: 62/2013). This study was carried out with the permission of all participating family members; informed written consent was obtained from the adult participants and from the parents/guardians/next-of-kin of children for collection of blood samples from the patients and non-affected parents as well as nasal biopsies.

Patients with a clinical picture of PCD were referred to clinics run by the Kuwait Ministry of Health for genetic assessment in collaboration with the Kuwait University Faculty of Medicine. The ethnic background of the patients included in this study include Asian Arabs descended from different tribes originating from the Arabian Peninsula (Al-Mutairi et al., 2022; Al-Mutairi et al., 2023). The inclusion criteria were clinical features typical of the hallmark disease of PCD (MIM: PS244400), including neonatal respiratory distress, chronic respiratory disease with symptoms of chronic airway infections, rhinosinusitis, otitis media, and bronchiectasis. Most patients were suspected to carry recessive disease-causing hereditary variants as more than one individual in their families exhibited similar clinical phenotypes.

Pedigrees for the families were constructed using Cyrillic version 2.1 (http://www.cyrillicsoftware.com/) as previously published (Al-Mutairi et al., 2022; Al-Mutairi et al., 2023). All radiological examinations of the patients in this study were performed at Sabah Hospital in Kuwait. The genomic DNA extractions and exome sequencing protocols were performed as described previously (Al-Mutairi et al., 2022; Al-Mutairi et al., 2023).

### Autozygosity mapping and variant screening

Genetic screening using autozygosity mapping was performed as described previously (Carr et al., 2013; Al-Mutairi et al., 2022; Al-Mutairi et al., 2023). AgileMultiIdeogram software was used to visualise the homozygous intervals using exome data for linkage, in which all the homozygous intervals were displayed against a circular ideogram for the 22 autosomal chromosomes for the related patients (Carr et al., 2013; Al-Mutairi et al., 2022; Al-Mutairi et al., 2023). Linkage analysis using autozygosity mapping was performed for all the affected individuals belonging to the same family, such as family KU-5 and family KU-9 together in one setting. This linkage was also performed for the patient belonging to family KU-2 separately. The reference genome annotation used was the Human Genome Build hg19 (UCSC genome browser) (Al-Mutairi et al., 2022; Al-Mutairi et al., 2023).

Primers for variant confirmation and segregation analyses were designed using the Primer3 software (http://frodo.wi.mit.edu/primer3/). Exon sequences were obtained from Genome Browser of the University of California, Santa Cruz (http://genome.ucsc.edu/) and RefSNP Report—dbSNP—NCBI (nih.gov) for all exons of *DNAH5* as follows. For the first heterozygous variant rs761622153 in family KU-2 exon 34, the forward primer sequence used was 5′-AAT​GAG​AAA​CGT​GAC​TTC​AAC​A-3´ and reverse primer sequence used was 5′-ACT​GGG​TAA​ATG​CAG​ATA​GTG​TC-3´. For the second heterozygous variant in family KU-2 exon 72, the forward primer sequence used was 5′-TTT​GAC​CTG​GAT​TAA​TTT​AAA​ATG​G-3´ and reverse primer sequence used was 5′-CAC​AAA​AGA​GGA​CCA​TCA​CC-3´. For the first homozygous variant in family KU-5 exon 73, the forward primer sequence used was 5′-TCA​GGG​ATT​TAC​AAA​AAT​GAT​TAC​T-3´ and reverse primer sequence used was 5′-TAC​GCC​AAA​GCT​AGG​AGG​TC-3´. For the second homozygous variant in family KU-5 exon 75, the forward primer sequence used was 5′-TGA​GTT​TGT​AGC​TTG​ACA​CCA​A-3´ and reverse primer sequence used was 5′-TCT​CCC​AAC​CGA​AGA​TAT​GC-3´. For the first heterozygous variant in family KU-9 exon 10, the forward primer sequence used was 5′-ATC​AAA​GGG​TTA​TTG​GTG​GAT-3´ and reverse primer sequence used was 5′-TCC​CAA​GGA​GGG​TAC​AAA​AA-3´. For the second heterozygous variant in family KU-9 exon 77, the forward primer sequence used was 5′-AAA​CAC​TTA​CGC​CAG​AAT​CCA-3´ and reverse primer sequence used was 5′-AGT​CGA​GCT​AGC​AGC​CTG​AG-3´. The Sanger sequencing protocol was performed as described previously (Al-Mutairi et al., 2022; Al-Mutairi et al., 2023), and the chromatograms were analysed using the GeneScreen software (Carr et al., 2011a; Al-Mutairi et al., 2022; Al-Mutairi et al., 2024)**.** The allele frequencies of the two homozygous missense variants of *DNAH5* in the Arab population were estimated using the allelic discrimination test described previously (Al-Mutairi et al., 2022; Al-Mutairi et al., 2023).

### *In silico* analyses of the two novel pathogenic variants in family KU-5

The algorithms used to estimate the pathogenicity of the two missense variants, *DNAH5*:c.12614G>A and *DNAH5*:c.12947T>C, in Tables 1, 2 are derived from the extended information in the MutPred database, which estimates the pathogenicities of the identified variants based on the protein sequences and model changes with regard to the structural features and functional sites between the wild-type and mutant sequences (Li et al., 2009). All pathogenicity scores mentioned in Table 3 are from the dbNSFP v4.1a database, which is a human database of aggregated scores from a large number of precalculated scores and algorithms that have also been augmented with custom rank scores to improve comparability. The dbNSFP v4.1a database focuses on non-synonymous single-nucleotide variants (nsSNVs) (Liu et al., 2020).

**TABLE 1:** Summaries of the *in silico* analyses for *DNAH5*:c.12614G>A showing the prediction tools with the most significant pathogenicity scores.

Algorithm	*DNAH5*:c.12614G>A
Loss of catalytic residue	at Trp 4206; *p* = 0.1006
Loss of helix score	*p* = 0.2271
Gain in ubiquitination	at Lys 4200; *p* = 0.0597
Loss of sheet	*p* = 0.0817
MoRFs binding	*p* = 0.2355
Loss of stability	*p* = 0.0169

**TABLE 2:** Summaries of the *in silico* analyses for *DNAH5*:c.12947T>C showing the prediction tools with the most significant pathogenicity scores.

Algorithm	*DNAH5*:c.12947T>C
Loss of catalytic residue	at Leu 4316; *p* = 0.01
Loss of helix score	*p* = 0.3949
Loss of stability	*p* = 0.0169
Loss of loop	*p* = 0.2897
Gain of disorder	*p* = 0.0154

**TABLE 3:** Summaries of the *in silico* analyses using nine meta-tools available in the dbNSFP database for the two newly identified missense variants *DNAH5*:c.12614G>A and *DNAH5*:c.12947T>C.

Algorithm	*DNAH5*:c.12614G>A	*DNAH5*:c.12947T>C
Mutation AAE	G4205E	L4316P
MetaLR	0.80713	0.68344
MetaSVM	0.88451	0.82694
REVEL	0.8933	0.92316
DANN	0.81213	0.95328
MutPred	0.99429	0.9549
MutationTaster	0.81001	0.81001
Provean rank	0.9587	0.92173
Polyphen-2	1	1

The results show that both variants have significant pathogenicity scores, indicating that both variants are deleterious and disease-causing variants.

### IF analyses of the nasal biopsies

**FIGURE 1:**
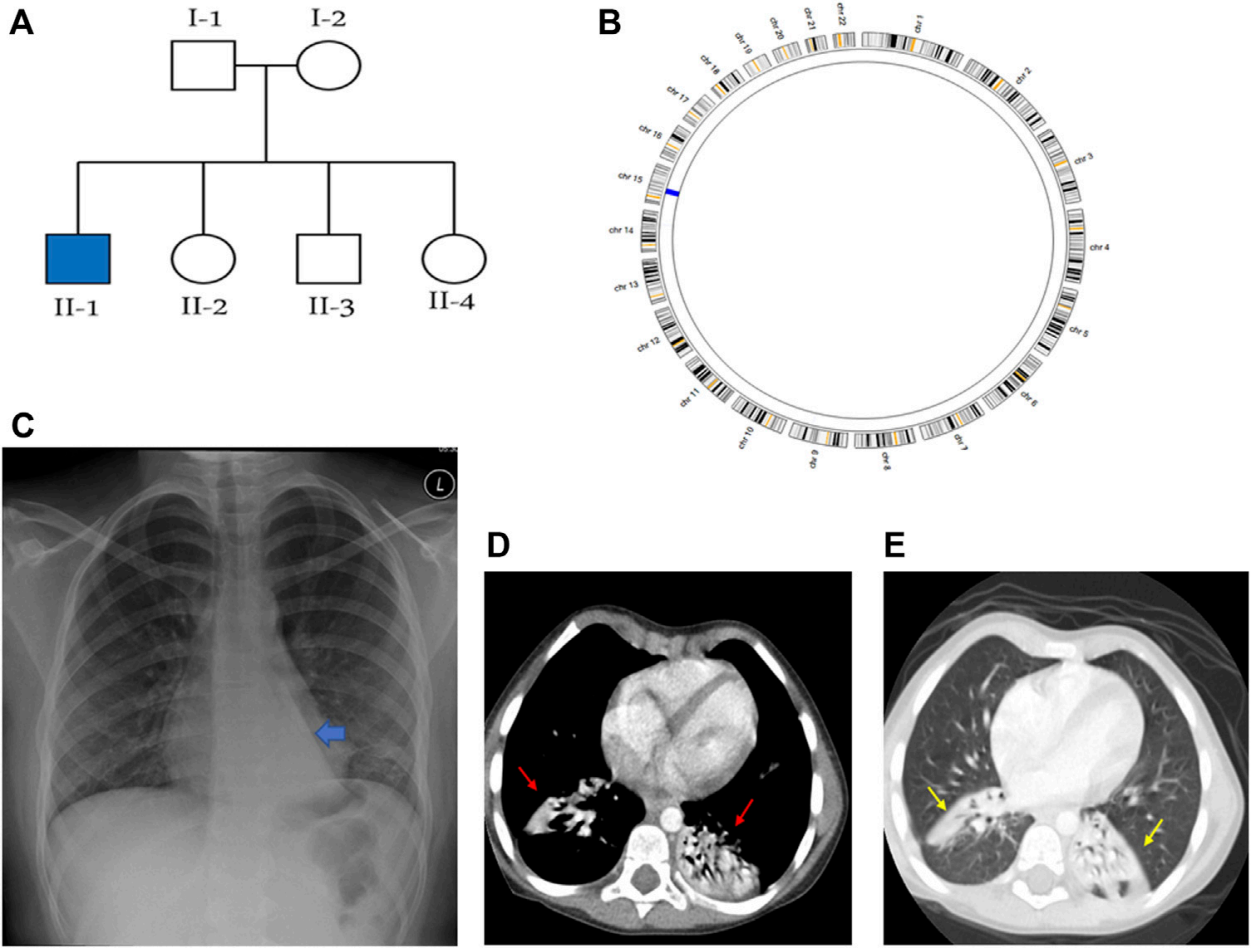
Summaries of the genetic and radiological data of family KU-2, where **(A)** shows the pedigree of family KU-2 and **(B)** shows the AgileMultiIdeogram for the KU-2.II.1 patient indicating that there was a single region of homozygosity (ROH) at chromosome 15. **(C)** Shows the chest X-ray for the KU-2.II.1 patient demonstrating a triangular retrocardiac opacity with a ‘flat-waist sign’ suggesting left lower lobe collapse. **(D, E)** Selected axial sections of the CT chest study of the same patient in the soft-tissue and lung windows showing subsegmental right lower lobe collapse in addition to the left lower lobe collapse (arrows).

Respiratory epithelial cells were obtained from the subjects by nasal brush biopsy and suspended in a cell culture medium. Samples were then spread onto glass slides, air dried, and stored at −80°C until use. The respiratory epithelial cells from the healthy control and five PCD-affected individuals highlighted in the pedigrees (Figures 1A, 3A, 6A) were dual labelled for IF analysis with antibodies against axonemal components (visualised with red-fluorescent secondary antibody) and antibodies to acetylated anti-tubulin (visualised with green-fluorescent secondary antibody) as the ciliary markers. The nuclei were stained with Hoechst 33342 (blue fluorescent) (Wallmeier et al., 2014; Al-Mutairi et al., 2023). The IF images were acquired with a Zeiss LSM 800 confocal microscope and processed using ZEN and ImageJ software (Al-Mutairi et al., 2022; Al-Mutairi et al., 2023).

The panel of primary antibodies used for the initial screening included mouse monoclonal anti-tubulin (acetylated) antibody (Sigma Aldrich, T6793) for staining the ciliary microtubules and rabbit polyclonal antibody anti-DNAH5 (1:300) (HPA037470, Atlas Antibodies). The secondary antibodies used were highly cross absorbed, including Alexa Fluor 488-conjugated goat antibodies to mouse (1:1,000) (A11029, Molecular Probes, Invitrogen) and Alexa Fluor 546-conjugated goat antibodies to rabbit (1:1,000) (A11035, Molecular Probes, Invitrogen) (Al-Mutairi et al., 2022; Al-Mutairi et al., 2023).

### TEM analyses of the nasal biopsies

For the TEM analyses, nasal biopsies were obtained from the middle turbinate for the same five affected individuals. The ciliated cells were first fixed with glutaraldehyde (2.5%) in Sorensen’s phosphate buffer (pH 7.4), following which the samples were treated with osmium tetroxide (1.3%), dehydrated using graded ethanol series, and immersed in hexamethyldisilazane as the chemical drying reagent. The samples were next embedded overnight in 1,2-epoxypropan-epon- mixture (1:1) at 4°C. After polymerisation, several sections were retrieved onto copper grids and were stained with Reynold’s lead citrate. TEM was then performed using the Philips CM10 device (Wallmeier et al., 2014; Al-Mutairi et al., 2022; Al-Mutairi et al., 2023).

## Results

We present the genetic and functional analyses for the five PCD individuals belonging to three families KU-2, KU-5, and KU-9 who were independently diagnosed with PCD from a cohort of 105 PCD individuals recruited from different hospitals in Kuwait. According to the medical reports of the patients, the paediatric pulmonologists performed differential diagnoses for all five patients and ruled out other conditions, such as cystic fibrosis, asthma, immunodeficiencies, and anatomic anomalies. These patients showed similar signs and symptoms as other healthy children, except for recurrent chest infections. The five patients were hospitalised during infancy for recurrent mild respiratory distress and required oxygen administration. None of the patients in this study required surgical intervention for bronchiectasis.

### Clinical and genetic findings for the KU-2.II.1 patient

The KU-2 family had one affected male child KU-2.II.1 who met the inclusion criteria for PCD symptoms with situs solitus (Figure 1A); the child underwent spirometry testing at various ages and showed declining pulmonary functions with both obstructive and restrictive patterns at the age of 18 years. His diagnosis of PCD was confirmed by genetic and *in vitro* functional analyses of the airway cilia. The selected chest X-ray for KU-2.II.1 shows a triangular retrocardiac opacity with ‘flat-waist sign’ suggesting left lower lobe collapse (Figure 1C). Selected axial sections of the computed tomography (CT) chest study for this patient from the soft-tissue and lung windows show subsegmental right lower lobe collapse in addition to the left lower lobe collapse (arrows in Figures 1D, E).

### Exome data reveals novel deleterious heterozygous variant of *DNAH5* in family KU-2

**FIGURE 2:**
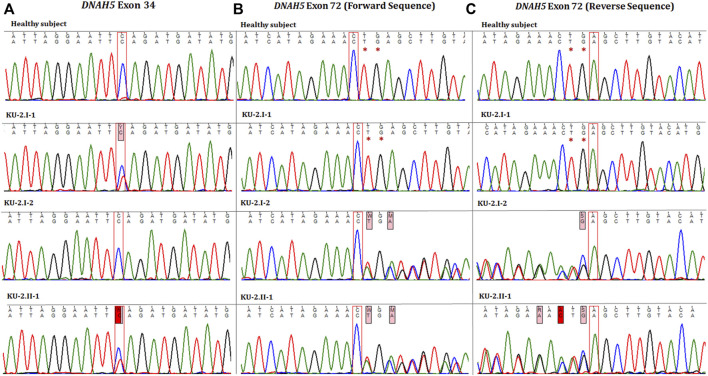
Chromatographs of the *DNAH5* variants for the members of family KU-2. **(A)** Represents the sequences of the healthy subject, parents (KU-2.I.1 and KU-2.I.2), and KU-2.II.1 patient, respectively. The KU-2.II.1 patient carried a heterozygous loss of function (LOF) variant (c.5503C>T; p.Gln 1835*; rs761622153) in exon 34 of *DNAH5* that predicted premature termination of translation. Segregation analysis demonstrated that the rs761622153 variant had a paternal inheritance. **(B, C)** Represent the forward and reverse sequences for the healthy subject, parents (KU-2.I.1 and KU-2.I.2), and KU-2.II.1 patient, respectively. The KU-2.II.1 patient carried a heterozygous novel LOF TG deletion mutation (c.12357_12358delTG; p.Glu4120Alafs*4) in exon 72 of *DNAH5* that also predicted premature termination of translation. Segregation analysis demonstrated that the variant (c.12357_12358delTG) had a maternal inheritance. This set of data indicates that the KU-2.II.1 patient has PCD due to inheritance of two LOF heterozygous pathogenic variants (non-sense and truncated).

Autozygosity mapping for KU-2.II.1 revealed a single region of homozygosity (ROH) summation at chromosome 15, as shown in Figure 1B. Filtering the variants in the exome data across this interval did not show any pathogenic variants; furthermore, since the parents are not related, this indicates that the PCD-causing variants are more likely inherited in compound heterozygous form. The analysis of exome data for deleterious variants in the known 53 PCD genes revealed that the patient carried two deleterious variants of *DNAH5* inherited in the compound heterozygous fashion. The first heterozygous variant was a heterozygous non-sense mutation NM_001369.2(DNAH5):c.5503C>T; (p.Gln 1835*); rs761622153 in exon 34 from paternal inheritance; RS = 5.8 and RVIS = 0.18 indicate that this is significantly damaging. This was recently reported as a PCD-causing variant inherited in a consanguineous family from United Arab Emirates (Alsamri et al., 2021). The second heterozygous variant was a novel frameshift deletion NM_001369.2(DNAH5):c.12357_12358delTG; (p.Glu4120Alafs*4) resulting in premature termination of translation from maternal inheritance. Segregation analysis confirmed that the two heterozygous variants of *DNAH5* were segregated with the disease phenotype, consistent with both being the PCD-causing variants in the KU-2.II.1 patient (Figures 2A–C).

### Clinical and genetic findings in the two PCD siblings of family KU-5

**FIGURE 3:**
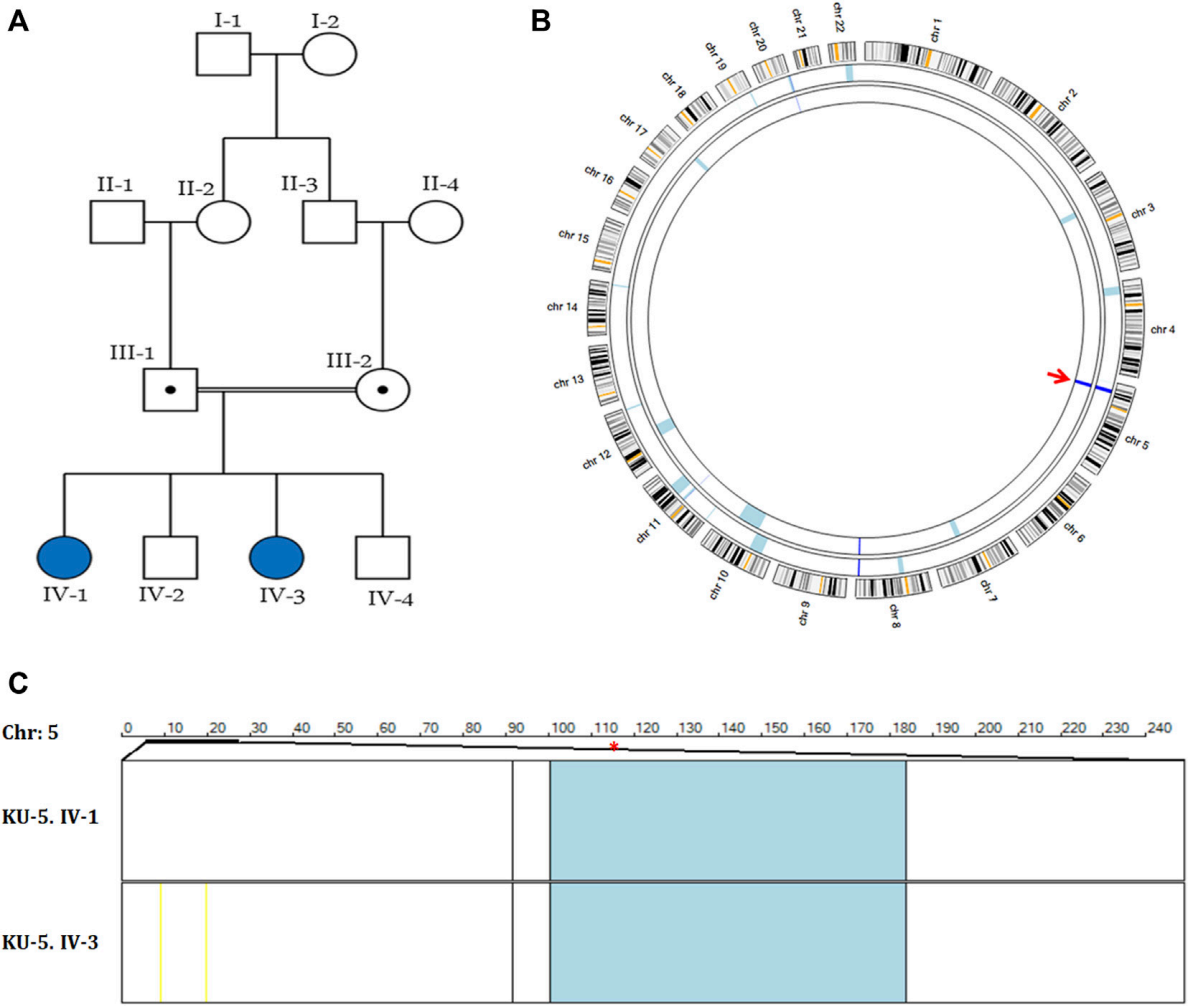
Genetic linkage data of family KU-5. **(A)** Pedigree of family KU-5 showing that the family has two affected females. **(B)** AgileMultiIdeogram showing the exclusive ROHs for KU-5.IV.1 and KU-5.IV.3 PCD individuals. The two PCD individuals had shared regions of autozygosity (dark blue) across the *DNAH5* locus on chromosome 5, as indicated by the red arrow. **(C)** Multiple linkage analysis at chromosome 5 for the two affected siblings using AgileVCFMapper software. The shared IBD interval at chromosome 5 was estimated to be (chr5:11,427,782-21,860,252), with a length of almost 10.5 kB. The physical location of *DNAH5* is at chr5:13,690,328-13,944,688 (within the shared IBD interval) and is highlighted by the red asterisk. As seen, each row represents the genotypes for chromosome 5 for one affected individual. The homozygous discordant alleles are represented as yellow lines, concordant autozygous alleles are represented as blue lines, and rare concordant autozygous alleles are represented as black lines.

**FIGURE 4:**
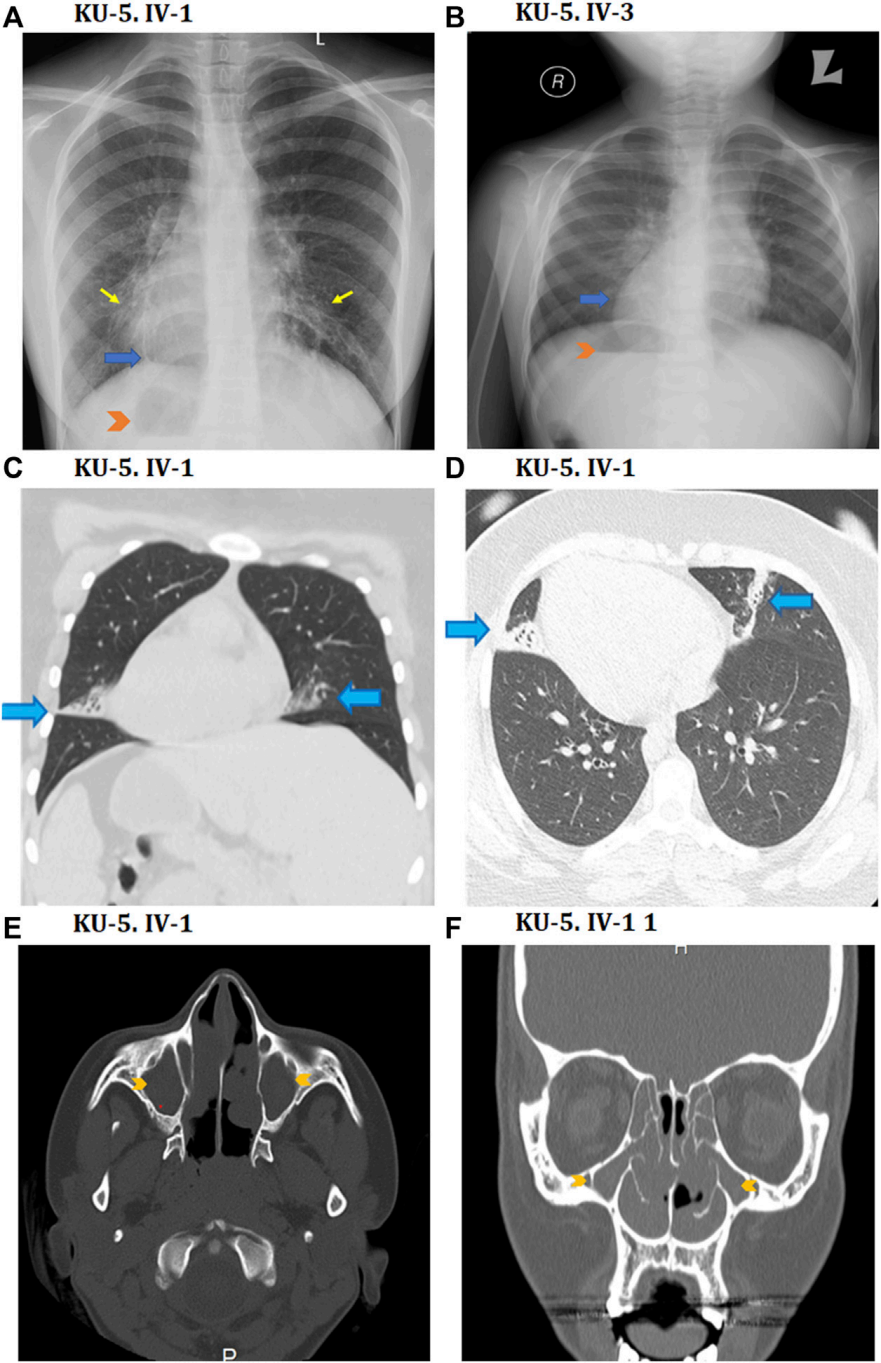
Radiological findings for the two PCD individuals of family KU-5. **(A, B)** Show chest X-rays for the KU-5.IV.1 and KU-5.IV.3 patients demonstrating right-sided heart apices (blue arrows) and gastric air bubbles (arrowheads) in conformity with situs inversus totalis. In addition, **(A)** shows mild diffuse bronchiectatic changes in the medial aspects of both lower zones (yellow arrows) for the KU-5.IV.1 patient. Selected coronal CT image in the lung window **(C)** as well as axial image of the lung window **(D)** from the CT chest study of a patient reveal situs inversus and subsegmental atelectasis in both lungs with mild underlying bronchiectatic changes (arrows). Selected axial **(E)** and coronal **(F)** reconstruction images of the CT-PNS study on the KU-5.IV.1 patient reveal complete opacification of the bilateral paranasal sinuses (arrowheads) in conformity with chronic inflammatory pansinusitis.

The KU-5 family was a multiplex family with two affected females, KU-5.IV.1 and KU-5.IV.3, both of whom presented with the following symptoms: chronic productive cough, recurrent pneumonias, wheezing dyspnea, and constant nasal mucopurulent secretions with situs inverses totalis (Figure 3A). Confirmed diagnoses of PCD were made at the ages of 7 and 5 years, with both subjects having situs inversus totalis*.* According to the KU-5.IV.1 patient’s questionnaire, the symptoms became gradually progressive with age, and the results of spirometry tests showed that the patient had declining pulmonary function, with both obstructive and restrictive patterns at the age of 12 years. The radiological findings for the two affected siblings (KU-5.IV.1 and KU-5.IV.3) can be summarised as follows: chest X-rays demonstrate right-sided heart apex (arrow) and gastric air bubbles (arrowhead), consistent with situs inversus totalis (Figures 4A, B). In addition, KU-5.IV.1 had mild diffuse bronchiectatic changes that were visible in the medial aspects of both lower zones, as highlighted by the thin yellow arrows (Figure 4A). The main radiological findings from the high-resolution CT (HRCT) chest examination of KU-5.IV.1 were situs inversus and subsegmental atelectasis in both lungs with mild underlying bronchiectatic changes (arrows in Figures 4C, D). Furthermore, selected axial and coronal reconstruction images of the CT of the paranasal sinus (CT-PNS) revealed complete opacification of the bilateral paranasal sinuses, consistent with chronic inflammatory pansinusitis (highlighted by arrows in Figures 4E, F).

### Autozygosity mapping reveals two novel homozygous variants of *DNAH5* in family KU-5

Autozygosity mappings for the two PCD individuals of family KU-5 showed a single shared interval that was identical by descent (IBD) at chromosome 5, as seen in Figure 3B. The estimated shared homozygous intervals were precisely determined using AgileVCFMapper software (chr5:11,427,782-21,860,252) and found to be almost 10.5 kB (Figure 3C). The *DNAH5* location at the shared IBD interval (chr5:13,690,328-13,944,688) is highlighted with a red asterisk in Figure 3C. The exome data for the two affected siblings were filtered at the shared IBD interval at chromosome 5 for screening of the deleterious variants of *DNAH5*.

**FIGURE 5:**
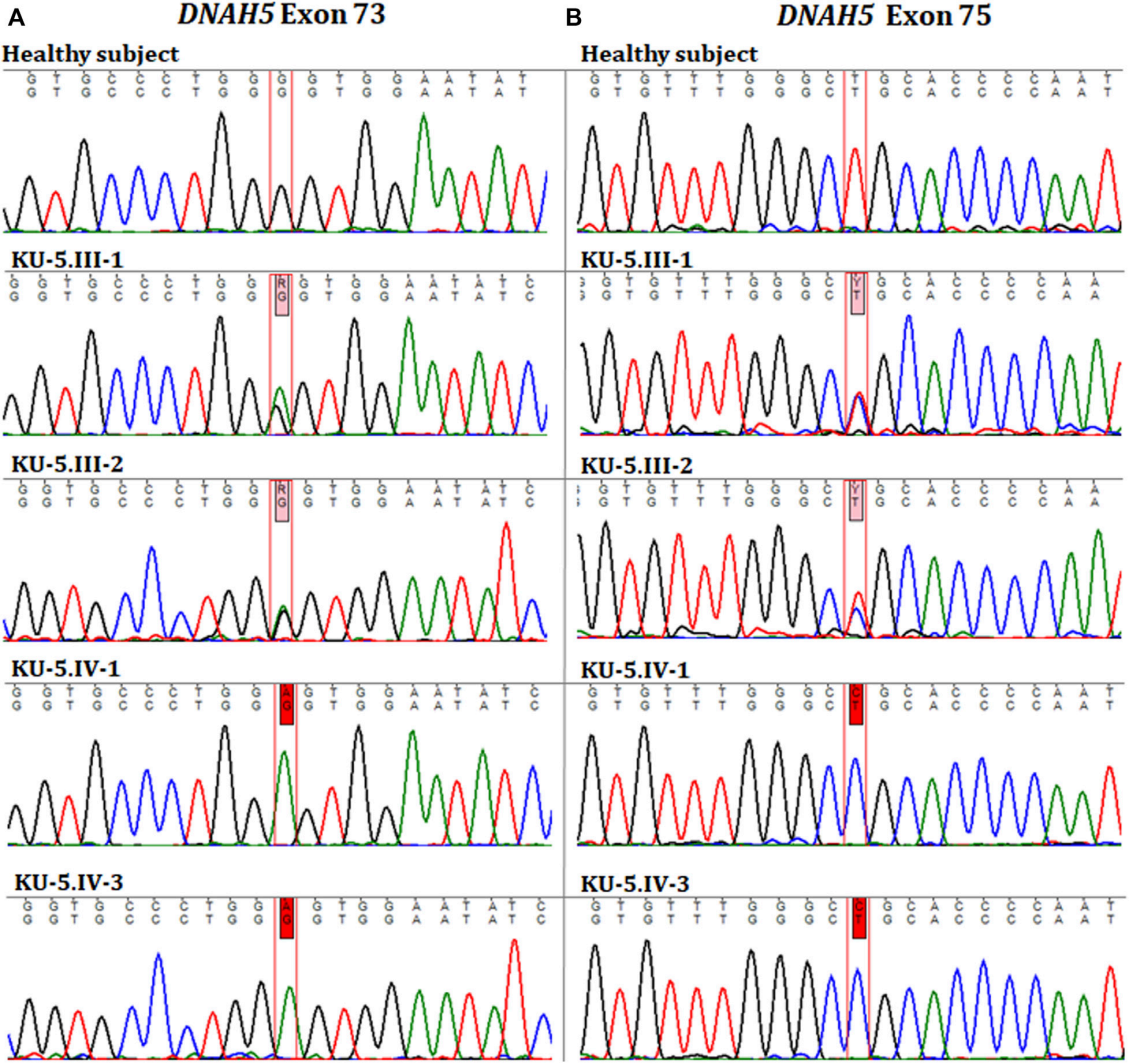
Chromatographs of the *DNAH5* variants in the members of family KU-5. **(A)** Represents the sequences for the healthy subject, parents (KU-5.III.1 and KU-5.III.2), and two PCD individuals (KU-5.IV.1 and KU-5.IV.3), respectively. The two PCD individuals inherited a novel homozygous missense variant in *DNAH5* (c.12614G>A; p.Gly4205Glu) located within exon 73 that results in a change of the amino acid glycine to glutamic acid. This variant has no RS number to date. The parents KU-5.III.1 and KU-5.III.2 were both carriers for this variant, consistent with it being a segregating and PCD-causing variant. **(B)** Represents the sequences for the healthy subject, parents (KU-5.III.1 and KU-5.III.2), and two PCD individuals (KU-5.IV.1 and KU-5.IV.3), respectively. Interestingly, the two PCD individuals inherited a second novel homozygous missense variant in *DNAH5* (c.12947T>C; p.Leu4316Pro) located within exon 75, which results in leucine to proline change. The second variant also has no RS number to date. The parents KU-5.III.1 and KU-5.III.2 were both carriers for the second variant, consistent with it being a segregating and PCD-causing variant. Genetic screening suggests that the two novel homozygous missense variants are responsible for developing PCD with situs inversus in family KU-5.

Two novel homozygous missense variants of *DNAH5* were identified in both patients by whole exome sequencing. The first homozygous missense variant NM_001369.2(DNAH5):c.12614G>A; (p.Gly4205Glu) was identified in exon 73, as shown in Figure 5A. The second homozygous missense mutation NM_001369.2(DNAH5):c.12947T>C; (p.Leu4316Pro) was identified in exon 75, as shown in Figure 5B. Both variants have no RefSNPs (rs) numbers to date in either Ensembl or the UCSC genome browsers. Following linkage and sequencing analyses, segregation analysis was performed to confirm that both variants identified in *DNAH5* were segregated with the disease phenotype. The results of segregation analyses confirmed that the parents, KU-5.III.1 and KU-5.III-2, were heterozygous carriers for both missense variants (Figures 5A, B). This implies that either of these variants could be responsible for PCD in family KU-5.

The two variants were assessed using different algorithms and statistical measurements to predict their pathogenicity. The first of these was rejected substitutions (RSs), defined as the number of substitutions expected under a neutral model minus the number ‘observed’ (estimated) for a particular alignment (Pazour et al., 2006; Al-Mutairi et al., 2022; Al-Mutairi et al., 2024); the second was polymorphism phenotyping (PolyPhen), which is a tool to predict the possible impacts of an amino acid substitution on the structure and function of a human protein (Adzhubei et al., 2013; Al-Mutairi et al., 2022; Al-Mutairi et al., 2024). The missense variant *DNAH5*:c.12614G>A resulting in an amino acid exchange (p.Gly4205Glu) had RS = 5.2, PPH = 3, MedPred = 81, and Varsome: likely pathogenic, all of which indicate that the variant is highly damaging. The missense variant *DNAH5*:c.12947T>C resulting in an amino acid exchange (p.Leu4316Pro) had RS = 4.6, PPH = 3, and MedPred = 83, all of which indicate that the second variant is also highly damaging. Together, these data for family KU-5 are unique and interesting since this is the only family in the study cohort that has two novel homozygous missense variants in *DNAH5* that are not reported previously in any database.

We extended our *in silico* analyses to predict the pathogenicities of the *DNAH5:*c.12614G>A and *DNAH5:*c.12947T>C variants. Our findings reveal noteworthy scores using mutation prediction tools designed to assess the pathogenicity of the identified mutations, as outlined in Tables 1–3. First, the loss of the catalytic residue is critical for ciliary targeting, and the loss of integrity of the catalytic domain in multi-ciliated respiratory cell mutants is directly related to ciliary mistargeting. This involves the targeting of cargo-containing membrane carriers or vesicles to the periciliary membrane (Lu and Madugula, 2018; Al-Mutairi et al., 2022; Cilleros-Rodriguez et al., 2022; Al-Mutairi et al., 2024). Loss of catalytic residues for the two missense variants were significantly low and were considered pathogenic (Tables 1, 2). In addition, the two variants *DNAH5:*c.12614G>A and *DNAH5:*c.12947T>C had significant loss of helix scores, indicating that both variants were highly damaging (Romero Romero et al., 2018). The missense variant *DNAH5:*c.12614G>A had significant scores for gain in ubiquitination at Lys 4200, loss of sheet, and loss of molecular recognition feature (MoRF) binding, all of which indicate that the variant is highly pathogenic (Table 1).

Furthermore, the missense variant *DNAH5:*c.12947T>C causes severe loss of the catalytic residue at Leu 4316, which is also directly associated with cilia mistargeting. The *DNAH5:*c.12947T>C variant leads to significant loss of stability, which is a common and widely used predictive tool for estimating the pathogenicity of missense mutations and disease development. The predicted pathogenicity from single amino acid changes affecting the thermodynamic stabilities of proteins highlights the role of protein stability in genetic diseases (Blaabjerg et al., 2023; Al-Mutairi et al., 2024). Furthermore, the *DNAH5:*c.12947T>C variant has a significant loss of loop score, which estimates the pathogenicity of the missense variant on a phosphate-binding loop (P-loop) motif that is necessary for ATP binding (Romero Romero et al., 2018; Al-Mutairi et al., 2024). It is well known that axonemal dyneins are directly involved in driving ciliary movements by providing the sliding forces for the microtubules within the cilia, which require energy from ATP hydrolysis within the heavy chain domains of axonemal dyneins (Mitchison and Mitchison, 2010). The *DNAH5:*c.12947T>C variant has a significant gain of disorder score supporting the previous pathogenic value of this amino acid substitution (Table 2).

In addition, we utilised nine meta-tools available in the dbNSFP database for annotation and functional predictions of all potential nsSNVs in the human genome (Liu et al., 2011; Kopanos et al., 2019; Liu et al., 2020; Al-Mutairi et al., 2022; Al-Mutairi et al., 2024). All of these determine the pathogenicity based on combined evidence from several other *in silico* predictors, and all of them predict both *DNAH5*:c.12614G>A and *DNAH5*:c.12947T>C to be deleterious/pathogenic and disease-causing missense variants, as seen in Table 3. Thus, the *in silico* analyses indicate that each variant is potentially highly pathogenic and PCD-causing, i.e. the presence of each variant alone may be sufficient for the development of PCD.

According to the ACMG criteria, the missense variant *DNAH5*:c.12614G>A has been annotated as likely pathogenic and missense variant *DNAH5*:c.12947T>C has been annotated to have uncertain significance despite their high pathogenicity prediction scores. Subsequently, allelic discrimination assays were performed on the two homozygous missense *DNAH5* variants using RT-PCR to estimate their carrier rate frequencies (*DNAH5*:c.12614G>A in Supplementary Figure S1 and *DNAH5*:c.12947T>C in Supplementary Figure S2) using a control panel composed of 100 DNA samples from normal individuals belonging to different Arabian tribes. As shown in Supplementary Figure S1, the genotype for the two PCD individuals is homozygous for the missense variant (A), and their parents are all heterozygous carriers for the wild-type allele (G) and defective allele (A), consistent with the Sanger sequencing data. Remarkably, the genotypes for the Arab control DNA panel show that all healthy individuals have the two wild-type alleles (G). Regarding the second variant, the genotype for the two PCD individuals is also homozygous for the second missense variant (C), and their parents are heterozygous carriers for the wild-type allele (T) and defective allele (C), which is again consistent with the Sanger sequencing data (Supplementary Figure S2).

### Clinical and genetic findings in the two PCD siblings of family KU-9

**FIGURE 6:**
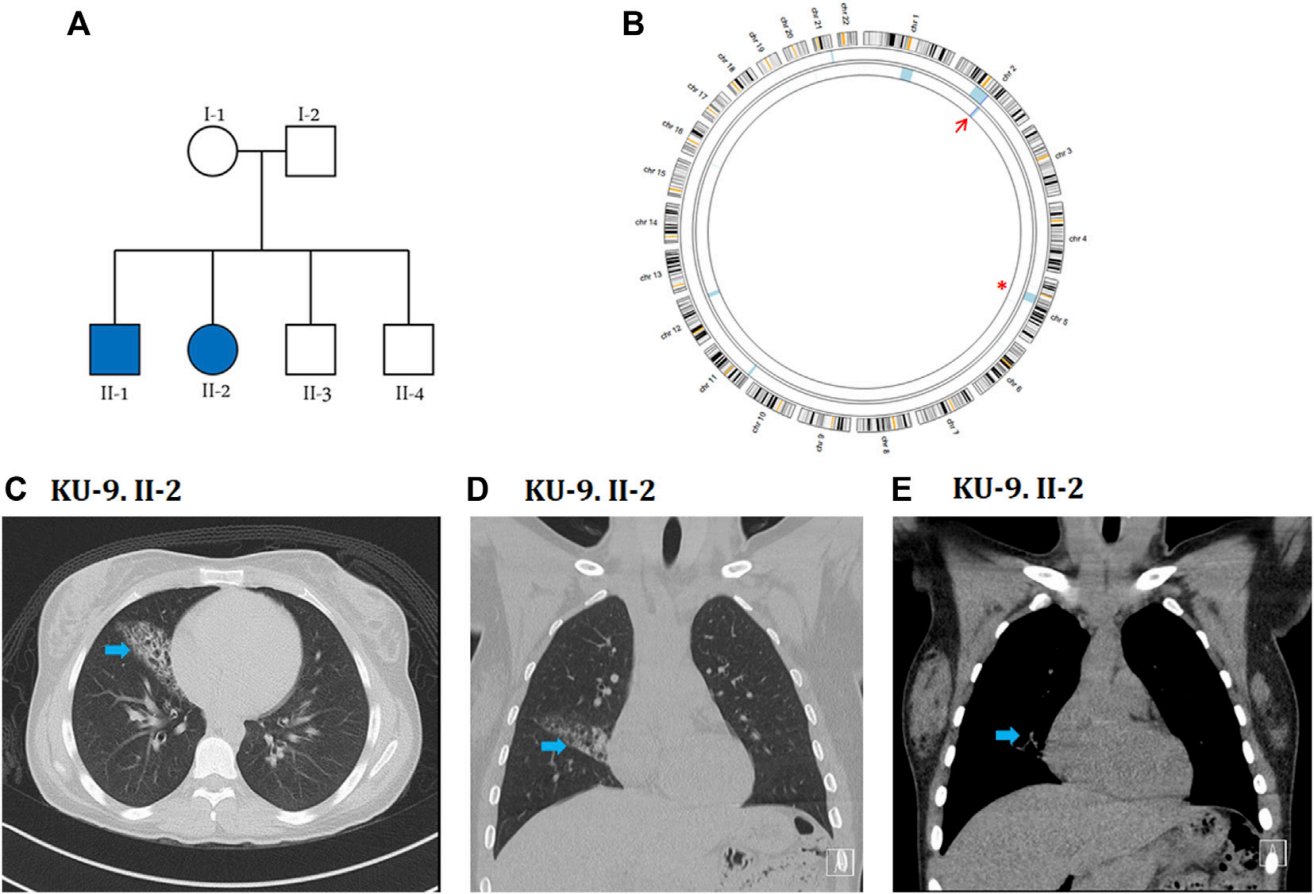
Genetic linkage and radiological data of family KU-9. **(A)** Shows the pedigree of family KU-9 that has two affected siblings. **(B)** AgileMultiIdeogram showing the exclusive ROH for the KU-9.II.2 patient (light blue) across the *DNAH5* locus at chromosome 5, highlighted by a red asterisk. There is a detectable shared IBD in chromosome 2 for the two siblings KU-9.II.2 and KU-9.II.1, as highlighted by the red arrow. Radiological data for the KU-9.II.2 individual are summarised in **(C–E)**; selected axial **(C)** and coronal **(D, E)** images of a HRCT study demonstrate situs solitus morphology with a wedge-shaped area of chronic inflammatory changes with bronchiectasis in the lateral segment of the right middle lobe (arrow). No other abnormalities or signs of active infection were noted. These abnormalities were stable in comparison to multiple previous studies over the years.

The KU-9 family is a multiplex family with two affected siblings KU-9.II.1 and KU-9.II.2 (Figure 6A) who met the inclusion criteria for PCD with situs solitus but showed only obstructive spirometry patterns. The male patient KU-9.II.1 was a smoker, so it is difficult to predict whether the pulmonary dysfunction was due to PCD or smoking; the patient was also substerile and had conceived two children by IVF. The female patient KU-9.II.2 had typical PCD symptoms, and her physician referred her to a genetic clinic for a confirmed diagnosis; she also experienced fertility problems and conceived a child by IVF after 5 years of marriage. The confirmed diagnoses of PCD were made through genetic and ciliary functional analyses of the two affected siblings KU-9.II.1 and KU-9.II.2 at the ages of 32 and 27 years, respectively. The main radiological findings from the HRCT chest investigation for KU-9.II.2 demonstrate situs solitus morphology with moderate cystic bronchiectatic changes involving the lateral segment of the right middle lobe. No other abnormalities or signs of active infection were observed (Figures 6C–E).

### Exome data reveals novel deleterious heterozygous variants of *DNAH5* in family KU-9

Although family KU-9 is a multiplex family with two affected siblings, the parents were not close relatives; it is very rarely the case that a family with two affected individuals has unrelated parents. However, exome sequencing of the two siblings indicated that the family was highly inbred, with the ROH summation being 220 and 117 for KU-9.II.1 and KU-9.II.2, respectively. The ROH summation cutoff value for inbreeding is 100, so the individuals theoretically belong to a highly inbred family. It is worth noting that family KU-9 belongs to a tribe that has practised consanguineous marriages among members of the tribe for generations and is considered to be particularly isolated, i.e. they have never married outside the tribe for historical and/or cultural reasons. Linkage analysis for the two affected siblings showed multiple ROH segments consistent with the ROH scores. However, none of these were shared between the two affected siblings, except for a minute interval at chromosome 2 (Figure 6B). Screening of this interval does not show any pathogenic variants. Subsequently, two interesting ROH intervals at chromosome 1 for patient KU-9.II.1 and chromosome 5 for patient KU-9.II.2 were prioritised for screening of the pathogenic variants. The ROH highlighted with a red asterisk (Figure 6B) is across the *DNAH5* locus at chromosome 5 for patient KU-9.II.2. The other ROH interval at chromosome 1 did not harbour any pathogenic variants. Interestingly, the exome data for the two affected siblings showed that both had two pathogenic heterozygous variants of *DNAH5.* Theoretically, autozygosity mapping can only map an IBD interval harbouring homozygous variants, and since we mapped the compound heterozygous variants in *DNAH5* in the two patients of family KU-9, this might explain the reason for not detecting the shared IBD using exome data across the *DNAH5* locus in the two affected siblings. The set of genetic data for family KU-9 is also unique and interesting since this multiplex family has two affected individuals who have inherited two novel compound heterozygous variants of *DNAH5* causing PCD with situs solitus, which has not been reported previously.

**FIGURE 7:**
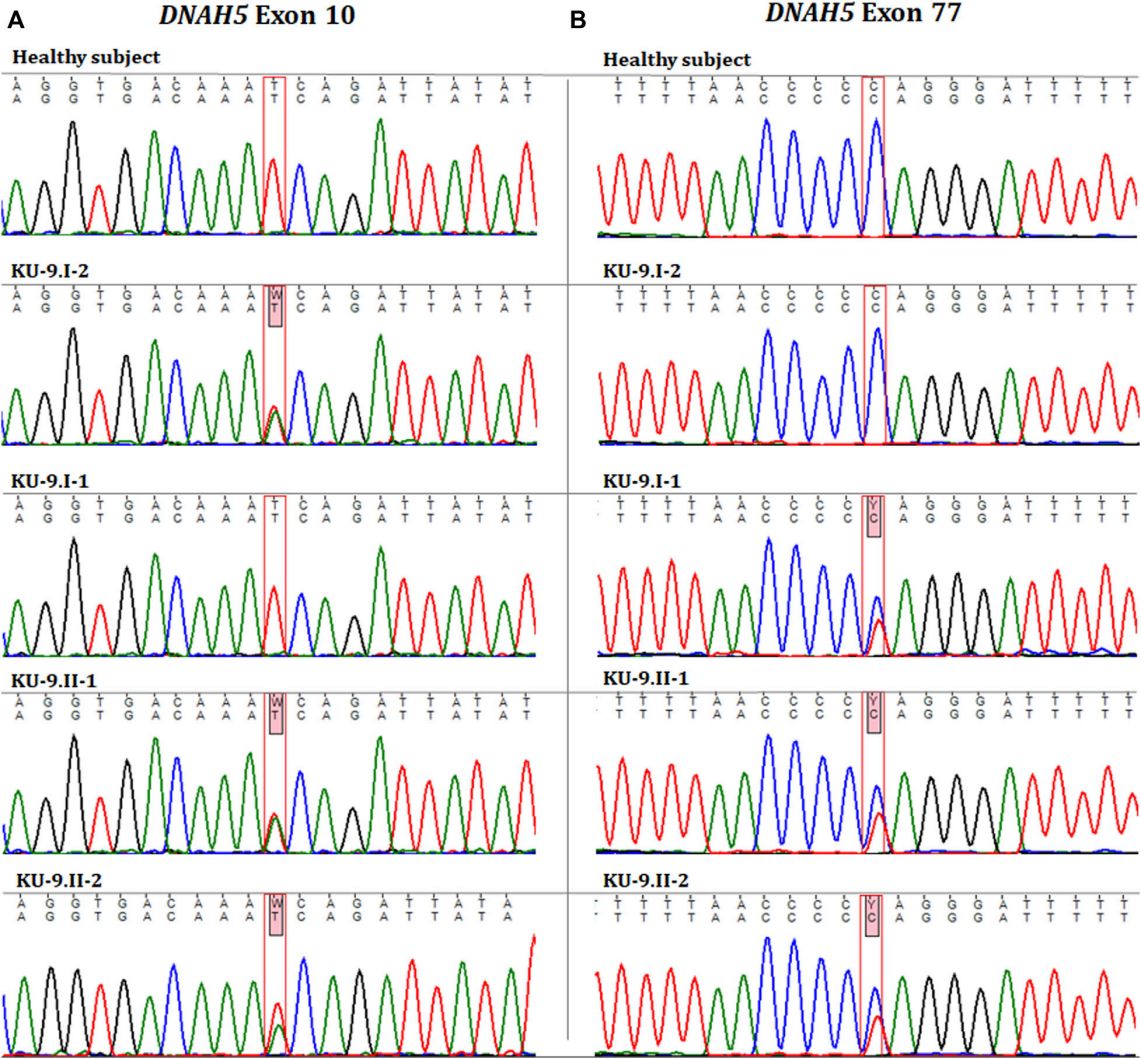
Chromatographs for the *DNAH5* variants in the members of family KU-9. Sequences for the healthy subject, parents (KU-9.I.1 and KU-9.I.2), and two PCD individuals (KU-9.II.1 and KU-9.II.2) are shown. The two PCD individuals inherited two compound heterozygous variants of *DNAH5*. **(A)** Shows that both PCD individuals were carriers for a missense variant leading to amino acid exchange (c.1206 T>A; p.Asn402Lys; rs140782270) in exon 10 of *DNAH5* that had a paternal inheritance. **(B)** Shows that both PCD individuals were carriers for a non-sense variant (c.13465C>T; p. Gln4489*) in exon 77 of *DNAH5* that leads to premature termination of translation and had a maternal inheritance. These data suggest that the two PCD individuals in family KU-9 had PCD due to inheritance of two heterozygous (missense and non-sense) pathogenic variants.

Exome and Sanger sequencings show that the two PCD individuals shared compound heterozygous variants; they were both carriers for a missense variant leading to amino acid exchange in exon 10 of *DNAH5*, i.e. NM_001369.2(DNAH5):c.1206 T>A; (p.Asn402Lys); rs140782270 (Figure 7A). The allele frequency of T was 100% for all ethnic backgrounds in the Ensembl genome browser with uncertain significance. They were also carriers for a second non-sense heterozygous variant in exon 77 of *DNAH5*, namely, NM_001369.2(DNAH5):c.13465C>T; (p. Gln4489*), that led to premature termination of translation and the variant having no RS number till date (Figure 7B). Segregation analyses for the two heterozygous variants showed that the heterozygous missense variant c.1206 T>A had a paternal inheritance. The heterozygous non-sense variant c.13465C>T had a maternal inheritance. This confirms that the presence of two heterozygous variants in the two PCD siblings of family KU-9 is most likely responsible for developing the PCD phenotype in the compound heterozygous mode of inheritance (Figures 7A, B). In conclusion, family KU-9 is the first multiplex family in which we failed to detect a shared IBD interval between the two affected individuals; nevertheless, both PCD individuals harboured the same heterozygous pathogenic variants. In conclusion, the variants mapped for *DNAH5* were pathogenic and associated with PCD. We also summarise the variants in the three Arabic families from Kuwait in Table 4.

**TABLE 4:** Summary of the *DNAH5* variants reported in this study in the three Arabic families in Kuwait.

Family	Patient	Gene variant	Amino acid change	RS number
KU-2	KU-2.II.1	c.5503C>T	p.Gln 1835*	RS761622153
		c.12357_12358delTG	p.Glu4120Alafs*4	No
KU-5	KU-5.IV.1	c.12614G>A	p.Gly4205Glu	No
	KU-5.IV.3	c.12947T>C	p.Leu4316Pro	No
KU-9	KU-9.II.1	c.1206T>A	p.Asn402Lys	RS140782270
	KU-9.II.2	c.13465C>T	p.Gln4489*	No

### Detection of ultrastructural defects of the motile cilia in the pulmonary system

**FIGURE 8:**
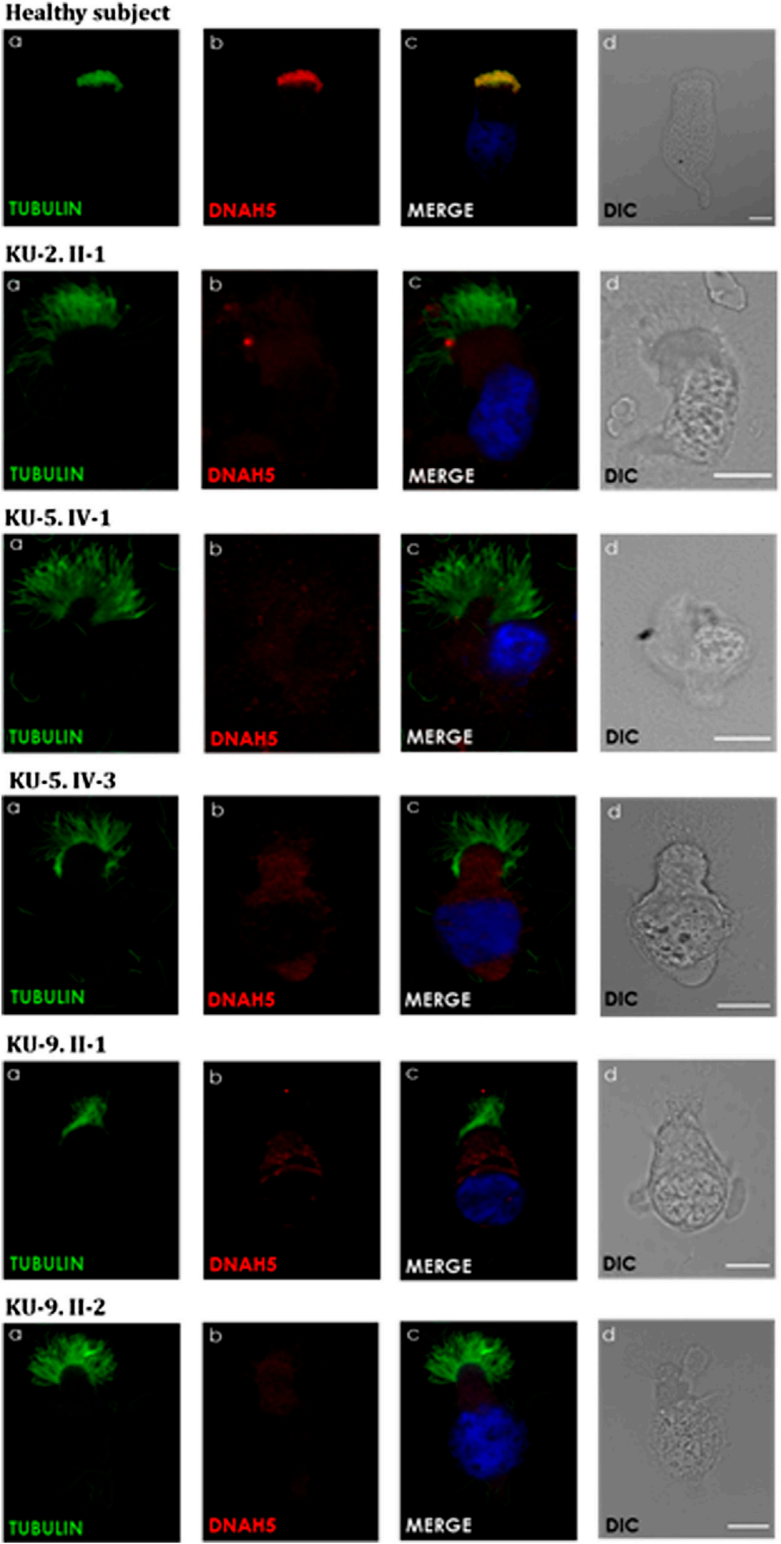
Immunofluorescence (IF) images of the primary airway epithelial cells of the PCD subjects. IF was performed using monoclonal anti-acetylated α-tubulin **(a)** and polyclonal anti-DNAH5 **(b)** antibodies for the five PCD individuals versus healthy controls. As seen in the controls, the merged images **(c)** show yellow co-staining within the ciliary axonemes, indicating that both proteins were co-localised within the airway cilia, compared to the images of the PCD individuals that show the absence of anti-DNAH5 staining. **(d)** shows differential interference contrast figures (DIC) for analyzed cells. Scale bar is 10 µm.

**FIGURE 9:**
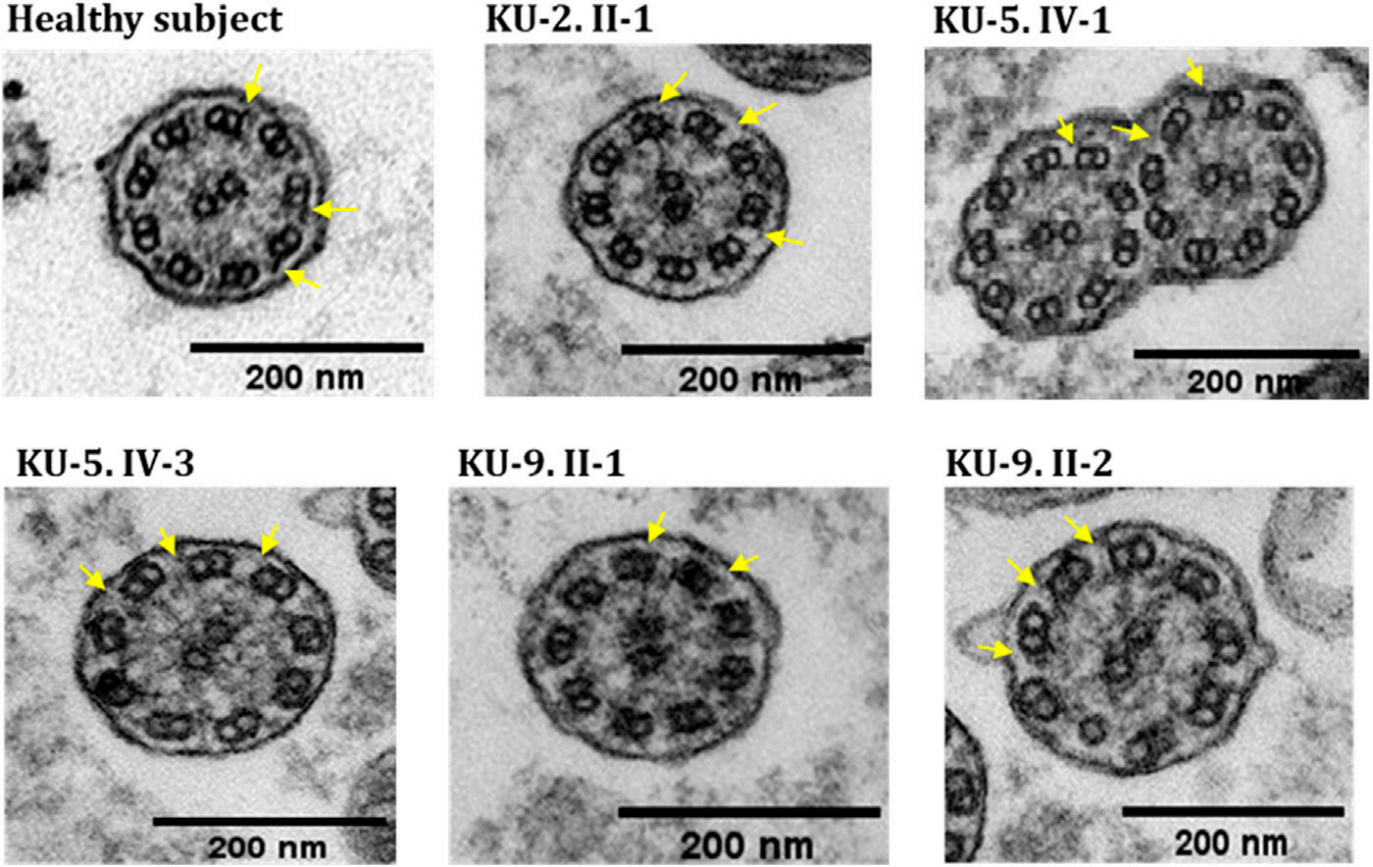
TEM sections of the ciliary axonemes for PCD individuals versus control. The TEM results for the ciliary axonemes of the ciliated epithelial cells of the respiratory system show that the PCD patients do not have ODAs as compared to the control, which is mainly seen in patients with defects in the genes encoding dynein arm components, consistent with their genotypes.

IF and TEM analyses were performed in this study on the nasal biopsies derived from five PCD individuals with different variants of *DNAH5* to identify the ultrastructural defects of the axoneme of the multi-ciliated respiratory epithelial cells. IF analyses of the primary airway epithelial cells using monoclonal antibodies showed staining for acetylated α-tubulin, which acts as an axonemal marker protein, in the five PCD individuals and in the healthy control, as seen in panel (a) of Figure 8. However, IF analysis using polyclonal antibodies directed against the ODA heavy chain DNAH5 showed complete lack of staining due to the pathogenic variants of *DNAH5*, resulting in loss of DNAH5 from the ciliary axoneme, as demonstrated by the absence of the DNAH5 signal in panel (b) of Figure 8 as well as absence of yellow co-staining in panel (c) of Figure 8 compared to the healthy subject who showed DNAH5 staining across the ciliary axoneme. In addition, TEM analyses confirmed the lack of ODAs (Figure 9), consistent with previously published reports (Olbrich et al., 2002; Hornef et al., 2006; Al-Mutairi et al., 2022). These analyses demonstrated that DNAH5 was absent from the ciliary axonemes of the affected individuals, thus supporting our genetic findings.

## Discussion

Our study presents novel findings regarding genetic variants within *DNAH5* inherited in both homozygous and compound heterozygous patterns, resulting in PCD with variable laterality defects. By employing an autozygosity mapping approach (Lander and Botstein, 1987; Abecasis et al., 2002; Carr et al., 2011b; Al-Mutairi et al., 2022; Al-Mutairi et al., 2023), we identified two novel homozygous missense variants within *DNAH5* in two PCD individuals from family KU-5 as well as compound heterozygous variants of *DNAH5* in PCD individuals from families KU-2 and KU-9, all of which were responsible for the development of PCD.

Previous studies on human *DNAH5* have characterised it as a structural component of the ODAs that are responsible for the main dynamic motility forces of the ciliary beats (Fowkes and Mitchell, 1998; Hornef et al., 2006; Al-Mutairi et al., 2022). The main structural components of the ODAs and inner dynein arms (IDAs) are motor heterotrimeric proteins that are preassembled in the cytoplasm prior to transportation into and assembly in the ciliary axonemes (Fowkes and Mitchell, 1998; Al-Mutairi et al., 2022). Human respiratory cilia have two distinct ODA heavy chains (HCs): ODA type 1, which is the component of the proximal part of the axoneme consisting of γ-HC DNAH5 and β-HC DNAH11; ODA type 2, which is the component of the distal part of the axoneme consisting of γ-HC DNAH5 and β-HC DNAH9 (Hornef et al., 2006; Loges et al., 2018; Al-Mutairi et al., 2022).

IF studies of the respiratory cilia show that DNAH5 has subcellular localisation along the entire lengths of the ciliary axonemes in both ODA type 1 and type 2 complexes. In addition, previous IF analyses of the airway cilia from PCD individuals with biallelic pathogenic *DNAH5* variants showed complete absence of not only DNAH5 but also DNAH11 (the ODA type 1 component) and DNAH9 (the ODA type 2 component). This shows that DNAH5 is required for accurate assembly of both type 1 and type 2 ODAs (Fliegauf et al., 2005; Schwabe et al., 2008; Pifferi et al., 2010; Knowles et al., 2012; Al-Mutairi et al., 2022).

Applying IF and TEM as two well-established *in vitro* functional analyses revealed the complete absence of the dynein arm protein component DNAH5 in all affected individuals in this study, indicating compromised axonemal assembly of both the proximal and distal localised ODA types 1 and 2 as well as lack of ODAs as the main ultrastructural defects in these individuals presenting with *DNAH5* pathogenic variants. These data support our genetic findings that previously unpublished homozygous and compound heterozygous *DNAH5* variants are the cause of PCD in these families and that the identified *DNAH5* variants are pathogenic.

## Conclusion

In previous reports, we described founder variants in *RSPH9* in eleven individuals shared by several unrelated Arabic families in our Kuwaiti PCD cohort (Al-Mutairi et al., 2023); we also reported novel founder variants in *DNAI2* in seven individuals (Al-Mutairi et al., 2022). Both of these are very rare PCD-causing genes in the global population. Herein, we identified the pathogenic variants of *DNAH5* in only five PCD individuals in our cohort of 105 Arabic PCD individuals recruited from different hospitals in Kuwait. Thus, in contrast to other ethnicities that have an incidence of almost 50%, *DNAH5* is not the most common PCD gene in the Kuwaiti population.

## Summary

In this study, we present our findings on five individuals having PCD belonging to three Arabic families, two of which are multiplex consanguineous families with two affected siblings each. All five PCD individuals had a history of neonatal respiratory distress, lifelong symptoms of wet cough, rhinosinusitis, otitis media, and bronchiectasis. Genetic screening revealed that these individuals had different pathogenic variants of *DNAH5* inherited in both homozygous and compound heterozygous forms. Molecular functional assessments using TEM and IF analyses confirmed the absence of the ODAs and missing DNAH5 components from the ciliary axonemes. In conclusion, *DNAH5* harbours rare novel pathogenic variants causing PCD in the Arab population of Kuwait.

## Data Availability

The raw sequence data is available on request, as it contains potentially identifiable information.

## Author’s note

The author contributions in detail: D.A-M. designed the study, provided the clinical data of the PCD individuals, collected DNA from all members of the PCD families, performed autozygosity mapping, variant screening and validation including *in silico* analyses, segregation analyses, IF analysis and interpretation, prepared all figures and wrote the manuscript. B.H.A. performed nasal brushing of all PCD subjects in the study. P.P. performed the TEM analysis. P.P. and H.O. reviewed the manuscript.
