# Supplementary material for: Novel pathogenic variants of DNAH5 associated with clinical and genetic spectra of primary ciliary dyskinesia in an Arab population
Source: Front Genet. 2024 Jul 10;15:1396797. doi: 10.3389/fgene.2024.1396797 (PMC11264286; doi:10.3389/fgene.2024.1396797)
Supplement: Supplementary file 1 [file Table1.DOCX]

**
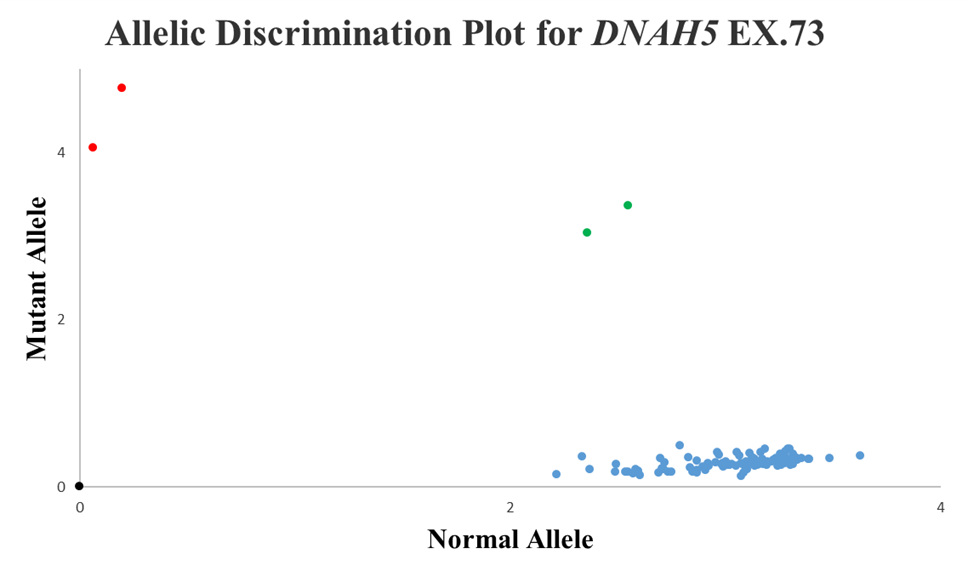
List of supplementary figures for *DNAH5* manuscript**

**Figure S1: Allelic discrimination Plot for c.12614 G>A variant in *DNAH5***. The two affected siblings showed homozygous mutant type pattern (AA; red dots) while their parents showed heterozygous mutant and normal alleles pattern (GA; green dots) consistent with Sanger sequencing data. A control DNA panel composed of 100 DNA samples collected randomly from healthy Arab individuals from different areas in Kuwait were run for the assay. The results showed all the control samples carry the wild type allele in homozygous pattern (GG; blue dots). This indicates that this mutation is very rare and the carriers for c.12614 G>A variant in *DNAH5* are hardly seen in Arabs.


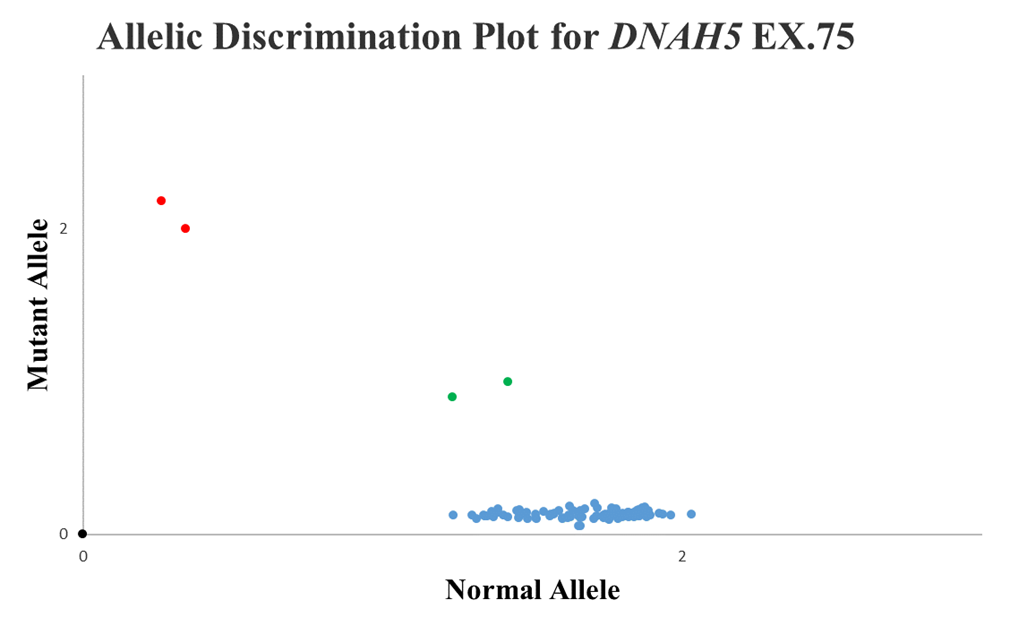


**Figure S2: Allelic discrimination Plot for c.12947 T>C variant in *DNAH5***. The two affected siblings showed homozygous mutant type pattern (CC; red dots) while their parents showed heterozygous mutant and normal alleles pattern (TC; green dots) consistent with Sanger sequencing data. A control DNA panel composed of 100 DNA samples collected randomly from healthy Arab individuals from different areas in Kuwait were run for the assay. The results showed all the control samples carry the wild type allele in homozygous pattern (TT; blue dots). This indicates that this mutation is very rare and the carriers for c.12947 T>C in *DNAH5* variant are hardly seen in Arabs.
